# Disseminated Herpes Simplex Virus Infection Presenting With Cytopenias in a Patient With Untreated Rheumatoid Arthritis

**DOI:** 10.1155/crdi/3786322

**Published:** 2026-10-12

**Authors:** Malvika Gupta, Christopher Graham

**Affiliations:** ^1^ Division of Hematology Mayo Clinic Rochester Minnesota USA mayo.edu; ^2^ Division of Hematology, Oncology and Transplantation University of Minnesota Minneapolis Minnesota USA umn.edu

**Keywords:** autoimmune disorders, cytopenia, herpes simplex virus, immunocompetent, mucositis, rheumatoid arthritis

## Abstract

**Background:**

Disseminated herpes simplex virus (HSV) infection is an uncommon but serious condition, typically seen in immunocompromised hosts. Cytopenias, including neutropenia and thrombocytopenia, are rare manifestations, particularly in patients not actively receiving immunosuppressive therapy.

**Case Presentation:**

We report a 75‐year‐old female with a history of rheumatoid arthritis (RA), previously on methotrexate and low‐dose prednisone, who presented with painful oral and genital ulcers, mucositis, and rapidly progressive bicytopenia (neutropenia and thrombocytopenia). Her recent brief reinitiation of methotrexate prompted evaluation for drug‐induced cytopenias; however, methotrexate levels were undetectable. Extensive infectious, nutritional, rheumatologic, and hematologic workup revealed HSV positivity from oral and inguinal ulcers. Bone marrow biopsy showed no evidence of hematologic malignancy or aplasia. She was treated with intravenous acyclovir, leading to clinical improvement and recovery of blood counts.

**Discussion:**

This case highlights the diagnostic complexity of cytopenias in patients with autoimmune diseases. While methotrexate toxicity and Felty’s syndrome were initially considered, the clinical trajectory and lack of supportive findings redirected focus to infectious causes. Disseminated HSV infection causing cytopenias is rare and often overlooked in immunocompetent or intermittently immunosuppressed patients. In this patient, immune dysregulation due to underlying RA and advanced age likely contributed to HSV dissemination and bone marrow suppression.

**Conclusion:**

Clinicians should maintain a high index of suspicion for disseminated HSV in patients with autoimmune diseases presenting with mucocutaneous lesions and cytopenias, even in the absence of significant immunosuppressive therapy. Prompt recognition and antiviral therapy are critical to prevent complications and improve outcomes.

## 1. Introduction

Herpes simplex virus (HSV) is a double‐stranded DNA virus belonging to the Orthoherpesviridae family, known for its capsid, which includes other human pathogens such as varicella zoster virus (VZV), Epstein–Barr virus (EBV), and cytomegalovirus (CMV). HSV is classified into two distinct types: HSV Type 1 (HSV‐1 or human herpesvirus‐1 [HHV‐1]) and HSV Type 2 (HSV‐2 or HHV‐2). Traditionally, HSV‐1 is associated with orolabial infections and HSV‐2 is associated with genital infections, although both types can cause disease at either site depending on the route of exposure. After primary infection, the virus establishes latency in sensory ganglia and can reactivate under certain conditions, particularly during periods of immunosuppression or impaired cellular immunity [1–3].

In most immunocompetent individuals, HSV infections remain localized and self‐limiting, and the occurrence of disseminated HSV infection is rare [4]. However, immunocompromised individuals, including those on chemotherapy, have active malignancies, primary or acquired immunodeficiency syndromes, severe diabetes mellitus, and autoimmune diseases or, on chronic immunosuppressive therapy, are at increased risk for reactivation and dissemination of HSV: affecting visceral organs such as the liver, lungs, or central nervous system, and in rare cases, the bone marrow [5–7].

One of the less recognized complications of disseminated HSV is bone marrow suppression, leading to cytopenias. To date, there are only two reported cases of cytopenias resulting from HSV infections [8, 9]. This presentation is typically reported in patients with profound immune compromise such as organ transplant recipients, chemotherapy recipients, or individuals with advanced HIV infection [5–7, 10, 11]. However, autoimmune diseases like rheumatoid arthritis (RA) may also predispose individuals to serious infections due to underlying immune dysregulation, even in the absence of active immunosuppressive therapy. There are a few case reports published of patients with an autoimmune disease such as RA presenting with disseminated herpes infection. However, the majority were on chronic immunosuppressive therapy for RA [12, 13].

In this report, we describe a rare case of disseminated HSV infection presenting as orogenital ulcers and rapidly progressive pancytopenia in an elderly female with RA, highlighting diagnostic challenges and the importance of considering HSV even in patients without overt immunosuppression.

## 2. Case Report

A 75‐year‐old African American female with a medical history of hypertension and RA, diagnosed approximately 30 months prior, presented to the emergency department with six days of painful oral ulcers and odynophagia. The patient reported a sudden onset of buccal ulceration (Figure 1), which severely limited her oral intake. She initially attributed these symptoms to the use of a newly introduced toothpaste, but the severity and persistence prompted evaluation.

**FIGURE 1:**
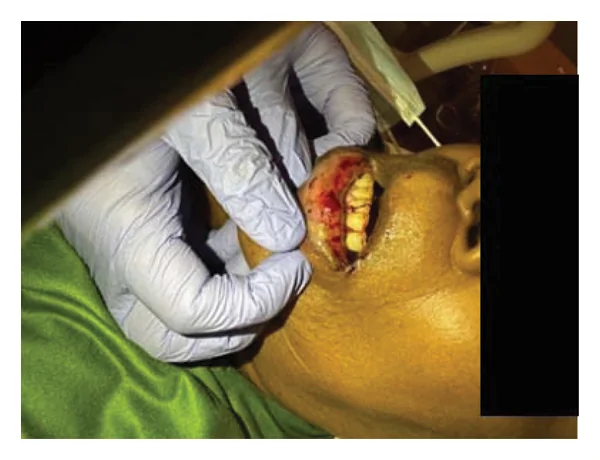
Buccal ulceration in the patient initially attributed to methotrexate toxicity, later confirmed to be due to herpes simplex virus infection.

Her prior treatment regimen for RA included methotrexate (15 mg weekly), folic acid, and low‐dose prednisone (5 mg daily). However, she had been lost to rheumatologic follow‐up for over a year. She discontinued methotrexate independently after experiencing intermittent, unexplained myoclonic movements, which she described as “jerking.” Over subsequent months, she experienced worsening inflammatory symptoms including decreased grip strength and progressive joint pain involving her hands, wrists, elbows, and shoulders, with intermittent involvement of her knees and ankles. She also reported paresthesia in both upper and lower extremities.

Approximately 2 weeks prior to presenting to the emergency room (ER), she developed progressive severe right shoulder pain. This continued for 11 days, and believing the symptoms to be a flare of her RA, she self‐initiated methotrexate at a reduced dose of 2.5 mg daily for 3 days, without folate supplementation. However, due to the new oral lesions limiting her oral intake, she came to the ER for evaluation.

Upon presentation, she denied systemic symptoms such as fever, fatigue, morning stiffness, Raynaud’s phenomenon, dry eyes, dry mouth, or photosensitivity. Vital signs were notable for hypertension (176/104 mmHg), and she appeared in moderate distress due to extensive oral mucosal involvement with tender, friable, erythematous, and ulcerated lesions over the hard palate. Musculoskeletal examination showed tenderness over bilateral proximal interphalangeal (PIP) and metacarpophalangeal (MCP) joints, along with chronic deformities consistent with advanced RA, including bilateral Boutonniere deformities of the fifth digits and limited range of motion of the right shoulder and lumbosacral spine. There was no evidence of splenomegaly or hepatomegaly. Dermatologic examination was notable for multiple hypopigmented lesions on the scalp (Figure 2), hyperpigmented, target shaped macules on the bilateral arms (Figure 3), and several painful ulcers localized to the bilateral inguinal regions. Additionally, a Stage I sacral decubitus ulcer was observed. The skin lesions were reported by the patient to have developed recently.

**FIGURE 2:**
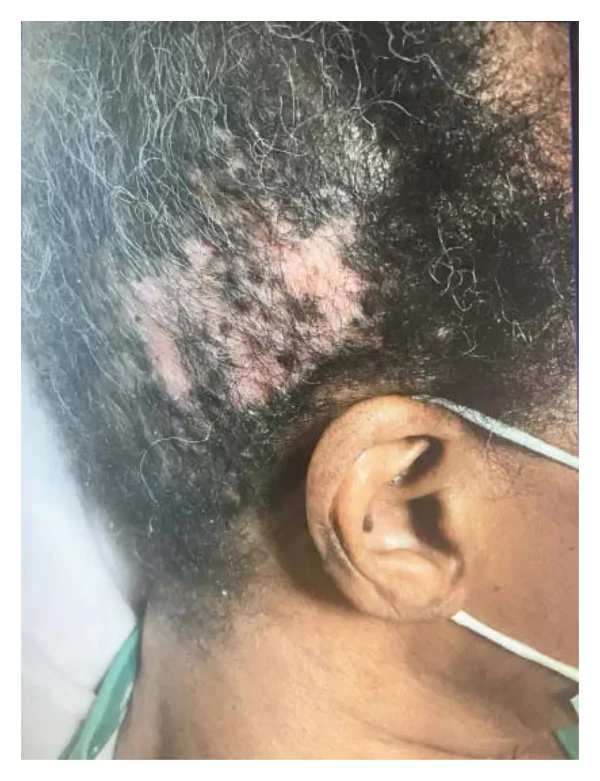
New onset hypopigmented lesions on the scalp attributed to disseminated herpes simplex virus infection.

**FIGURE 3:**
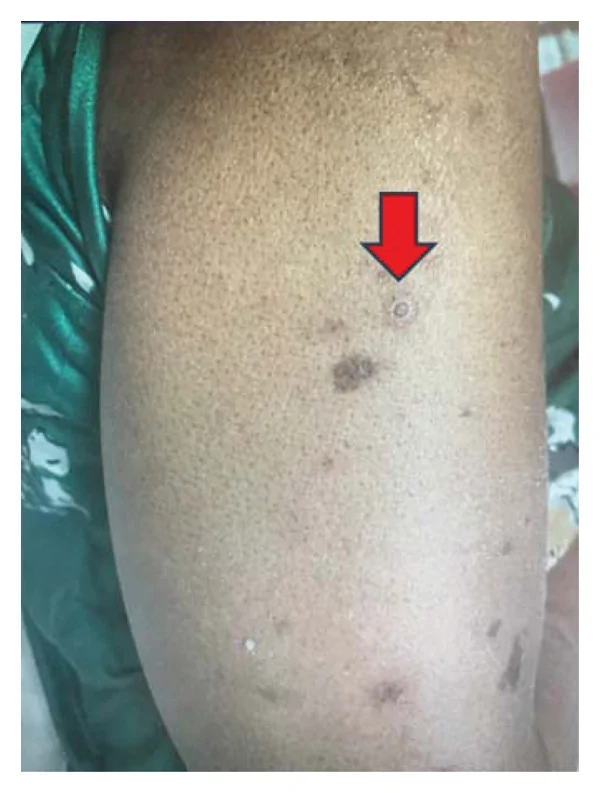
New‐onset hyperpigmented, target shaped lesions on the arms (indicated by the red arrow), clinically consistent with erythema multiforme due to disseminated herpes simplex virus infection.

Laboratory investigations (Table 1) revealed elevated inflammatory markers, with a C‐reactive protein (CRP) level of 52.64 mg/L and an erythrocyte sedimentation rate (ESR) of 43 mm/hr. Hematologic parameters were significant for mild thrombocytopenia (platelet count 109 × 10^9^/L), severe neutropenia (absolute neutrophil count 0.7 × 10^9^/L), and a normal hemoglobin level. Urinalysis revealed microscopic hematuria and proteinuria. Given the recent reexposure to methotrexate, serum methotrexate levels were obtained and returned undetectable (< 0.02 μmol/L), ruling out acute methotrexate toxicity. Nevertheless, she was empirically started on leucovorin rescue therapy, without any subsequent improvement in mucositis or cytopenias.

**TABLE 1:** Laboratory investigations at admission.

Inflammatory markers	
ESR (mm/hour)	43 (high)
CRP (mg/L)	52.64 (high)
Haptoglobin (mg/dL)	281 (high)
Procalcitonin (ng/mL)	0.13 (high)
Immunology	
ANA	< 1:40
Anti‐dsDNA (IU/mL)	< 10
Anti‐CCP IgG (U/mL)	> 150 (high)
RF quantitative (IU/mL)	> 360 (high)
Anti‐SSA (U/mL)	< 12
Anti‐SSB (U/mL)	< 12
Immunology (ctd.)	
Anti‐RNP (U)	< 1
Anti‐Smith (U/mL)	< 1
C3c (mg/dL)	159
C4c (mg/dL)	29
Nutritional parameters	
Vitamin B12 (pg/mL)	534 (normal)
Folate (ng/mL)	> 22.3 (normal)
Vitamin C serum (mg/dL)	0.1 (low)
Zinc plasma (mcg/dL)	52 (low)
Copper serum (mcg/dL)	166 (normal)
Therapeutic drug monitoring	
Serum methotrexate (μmol/L)	< 0.02

*Note:* Anti‐dsDNA: anti‐double‐stranded DNA antibody; anti‐SSA: anti‐Sjogren’s Syndrome Type A antibody; anti‐SSB: anti‐Sjogren’s Syndrome Type B antibody; anti‐RNP: anti‐ribonucleoprotein antibody; C3c: complement component 3c; C4c: complement component 4c.

Abbreviations: ANA: antinuclear antibody; anti‐CCP: anticyclic citrullinated peptide; CRP: C‐reactive protein; ESR: erythrocyte sedimentation rate; RF: rheumatoid factor.

Nutritional deficiency screening revealed low serum levels of zinc and vitamin C, while serum folate, vitamin B12, and copper levels were within normal limits (Table 1). A rheumatologic workup demonstrated a markedly elevated anticyclic citrullinated peptide (anti‐CCP) IgG level (> 150 U/mL) and rheumatoid factor (> 360 IU/mL), consistent with seropositive RA. Antinuclear antibodies (ANA), anti‐dsDNA, anti‐SSA, anti‐SSB, and RNP/Sm antibodies were negative. Complement levels and the free light chain ratio were within normal limits (Table 1). Serum protein electrophoresis revealed an acute‐phase reaction pattern with oligoclonal bands in the gamma region, and urine protein electrophoresis revealed tubular proteinuria without evidence of monoclonal gammopathy.

She was empirically started on intravenous Cefepime for methicillin‐susceptible *Staphylococcus aureus* (MSSA) and Gram‐negative coverage. Her sacral wound culture grew methicillin‐resistant *Staphylococcus aureus* (MRSA), and she was initiated on intravenous vancomycin, and cefepime was discontinued. Dermatology consultation was obtained for evaluation of the cutaneous lesions. The lesions on her arms were clinically consistent with erythema multiforme, while a skin biopsy demonstrated perivascular dermatitis.

Given the characteristic orogenital distribution of the ulcerations, testing for infectious etiologies were pursued (Table 2). PCR testing of both oral and inguinal lesions confirmed the presence of HSV‐1. Additional infectious workup, including HIV, *Treponema pallidum*, VZV, *Chlamydia trachomatis*, and *Neisseria gonorrhoeae* PCRs from lesion swabs, showed negative results.

**TABLE 2:** Infectious workup for orogenital lesions with bicytopenia in our case report.

	
Varicella zoster virus ulcer (PCR)	Negative
Varicella zoster virus blood (PCR)	Negative
Herpes simplex virus culture (blood)	Negative
Herpes simplex virus‐1 oral ulcer (PCR)	Positive
Herpes simplex virus‐1 sacral ulcer (PCR)	Positive
Herpes simplex virus‐2 oral ulcer (PCR)	Negative
Herpes simplex virus‐2 sacral ulcer (PCR)	Negative
Herpes simplex virus‐1 blood (PCR)	Positive
Herpes simplex virus‐2 blood (PCR)	Negative
Cytomegalovirus quantitative blood (PCR)	Not detected
Hepatitis C antibody serum	Nonreactive
*Treponema pallidum* DNA sacral ulcer (PCR)	Negative
IgG parvovirus serum	Positive
IgM parvovirus serum	Negative
Parvovirus quantitative blood (PCR)	Not detected
Epstein–Barr virus IgG capsid serum	Positive
Epstein–Barr virus IgM capsid serum	Negative
Epstein–Barr virus quantitative blood (PCR)	Not detected
HIV 1/2 Ag/Ab serum	Nonreactive
*Neisseria gonorrhoeae* DNA oral ulcer (PCR)	Negative
*Neisseria gonorrhoeae* DNA sacral ulcer (PCR)	Negative
Chlamydia trachomatis DNA oral ulcer (PCR)	Negative
Chlamydia trachomatis DNA sacral ulcer (PCR)	Negative
Culture for sacral wound	Positive for methicillin‐resistant *Staphylococcus aureus* (MRSA)

*Note:* IgG: immunoglobulin‐G; IgM: immunoglobulin‐M; DNA: deoxyribonucleic acid.

Abbreviations: HIV: human immunodeficiency virus; PCR: polymerase chain reaction.

Her platelet count nadired at 19 × 10^9^/L, and her absolute neutrophil count nadired at 0.01 × 10^9^/L, with associated 2% peripheral blasts and atypical lymphocytes on peripheral smear. Infectious workup for other marrow‐suppressive viruses also revealed positive EBV PCR and Parvovirus B19 IgG. Given the coinfection and worsening hematologic profile, further evaluation for marrow‐infiltrative and clonal hematologic disorders was undertaken. Peripheral blood flow cytometry identified a small atypical T‐cell population (CD4+, CD3−) of uncertain clinical significance but no evidence of a definitive lymphoid or myeloid neoplasm. Absolute counts for large granular lymphocytes (LGLs) were low (T‐LGL count 53/μL), arguing against LGL leukemia. Bone marrow aspiration and biopsy showed a hypercellular marrow with preserved trilineage hematopoiesis and no morphological or immunophenotypic features of leukemia, myelodysplastic syndrome, or aplastic anemia. Bone marrow flow cytometry was unremarkable.

Given the multifocal mucocutaneous involvement and systemic symptoms, disseminated HSV infection was suspected, and intravenous acyclovir was initiated at a dose of 4 mg/kg three times daily. The patient experienced gradual clinical improvement in her mucosal lesions and systemic symptoms following antiviral initiation and supportive care. The patient’s hematologic parameters gradually improved, with stabilization of platelet and neutrophil counts by day eight of therapy. Serum HSV PCR was drawn, which returned negative, suggesting clearance of infection. These findings supported the diagnosis of bone marrow suppression secondary to disseminated HSV infection, likely exacerbated by underlying immune dysregulation from RA and latent EBV coinfection.

## 3. Discussion

We present here an elderly female with prior history of advanced RA controlled on oral methotrexate, but self‐discontinued months prior to presentation, who presented with disseminated HSV lesions of the hard palate and inguinal region and cytopenias. The initial concern was for methotrexate toxicity, which was subsequently ruled out. Her hospital course was significant for rapidly progressive neutropenia, with associated thrombocytopenia, with improvement after initiation of intravenous acyclovir.

This case poses a particular diagnostic dilemma with multiple factors increasing the likelihood of bicytopenia (Table 3). Initially felt to be related to methotrexate‐induced toxicity, subsequent testing showed an undetectable level of methotrexate in the blood after a recent cumulative 7.5 mg dose. In patients with RA, Felty’s syndrome, which is a constellation of neutropenia, splenomegaly in the setting of untreated RA can be considered [14, 15]. However, in our case, there was no splenomegaly, and the progressive and profound neutropenia improved without direct therapy for RA. Other common causes of neutropenia associated with mucositis include nutritional deficiencies such as folate, copper and B12; however, these levels were normal prior to any replacement. Of note, though zinc and vitamin deficiencies can cause mucositis, they are not associated with neutropenia [16]. A more serious concern for neutropenia in the setting of myelophthisis is due to an infiltrative process in the bone marrow, either malignancy or infectious. However, her bone marrow biopsy showed no evidence of an infiltrative process or an aplastic process in the setting of aplastic anemia. In patients with rheumatologic diseases, large granulocytic leukemia, a clonal disorder of NK cells, can cause neutropenia but not thrombocytopenia [17].

**TABLE 3:** Summary of differential diagnosis of bicytopenia with mucositis.

S. no.	Differential diagnosis for bicytopenia with mucositis	Diagnostic reasoning (as to why this applies to our case)	Diagnostic reasoning (as to why this does not apply to our case)
1	Methotrexate toxicity	History of consuming a recent cumulative dose of 7.5 mg of methotrexate	1. Undetectable methotrexate serum level after a recent cumulative dose of 7.5 mg
			2. Leucovorin rescue did not improve the cytopenia or the mucositis
2	Felty’s syndrome	Felty’s syndrome is a constellation of neutropenia and splenomegaly in the setting of untreated RA	1. No splenomegaly
			2. Neutropenia improved without therapy for rheumatoid arthritis
3	Nutritional deficiencies (Vitamin B12, folate, copper, zinc, vitamin C)	A common cause of mucositis with or without cytopenias	1. Vitamin B12, folate, and copper levels were normal at baseline
			2. Zinc and vitamin C levels were low, which can explain the mucositis, but does not explain the cytopenias
4	Bone marrow infiltration	Bicytopenia in the setting of myelophthisis—due to an infiltrative process in the bone marrow, either malignancy or infectious	Bone marrow biopsy was normal
5	Large granulocytic leukemia	In patients with rheumatologic diseases, large granulocytic leukemia, a clonal disorder of NK cells, can cause neutropenia	Does not explain the concurrent thrombocytopenia

Finally, infectious‐mediated cytopenias were considered. To our knowledge, there have been only two case reports implicating herpes infection as a cause for rapidly progressive cytopenias, although primarily in an immunocompromised host [8, 9]. One case report describes a case of disseminated HSV‐1 infection causing pancytopenia in the setting of ESRD and diabetes with manifestations of aplastic anemia [9]. Another case report describes a patient with HSV‐2 infection presenting as isolated thrombocytopenia [8]. In our patient, the patient’s blood count improved with IV acyclovir therapy, supporting the diagnosis of disseminated herpes infection despite an immunocompetent state.

Some viral infections such as HIV, hepatitis viruses, EBV, and Parvovirus B19 are more likely to be associated with acquired pancytopenia [18, 19]. HHV‐8 and HHV‐6 have been reported to cause bone marrow failure in patients who have undergone bone marrow transplant, suggesting a causative effect of HHV family viruses in bone marrow suppression [19, 20].

Dissemination of herpes virus is rare and usually occurs in an immunosuppressed host [2, 3]. However, there are multiple case reports of seemingly immunocompetent hosts who developed serious disseminated HSV. Autoimmune diseases such as RA increase the risk of disseminated herpes virus infection due to dysregulation of the immune system [21]. Typically, this is in the setting of immunosuppressive medications such as steroids or disease‐modifying antirheumatic drugs (DMARDs) [22–26]. Our patient was taking intermittent immunosuppression; however, in patients with autoimmune diseases, low‐dose immunosuppressive drugs may trigger HSV reactivation in combination with other impaired immunity causes (immune dysregulation, advanced age) [26]. This is driven mostly by impaired cell‐mediated immunity. Both increased physiological stresses through cortisol release, and age‐related decreased cellular and humoral immunity can contribute to dissemination of HSV [27, 28].

Concurrent viral infections with EBV and HSV‐1 in the setting of impaired cellular immunity, as shown in one case, led to fulminant cytopenias and impaired cellular immunity due to hemophagocytic lymphohistiocytosis [29]. Our patient had concurrent EBV infection, likely adding to the thought that the combined viremia might play into cytopenias. However, further studies are required to determine whether EBV coinfection can lead to disseminated HSV.

The lack of clinical suspicion of disseminated HSV infection in seemingly immunocompetent hosts can lead to delayed diagnosis and, therefore, higher morbidity and mortality [30, 31]. All of these clinical scenarios raise concern for more awareness of serious disseminated HSV infection occurring in immunocompetent patients. Therefore, clinicians should be aware that even if the patient is immunocompetent, certain factors may still aid the reactivation and dissemination of the virus. The lesions may be generalized. However, the presence of the characteristic painful vesicular lesions may aid in diagnosis. Further studies are needed to determine the direct immunosuppressive and immune deregulatory effect found in rheumatologic disorders contributing to the risk of disseminated herpes virus infections.

## Funding

This research did not receive any specific grant from funding agencies in the public, commercial, or not‐for‐profit sectors to support this work.

## Consent

The patient described in this case report was unable to provide informed consent due to their clinical condition. Despite thorough efforts, no legally authorized representative, family member, or caregiver was available to provide consent. All identifying information has been removed to protect patient privacy, and no details that could reasonably lead to patient identification are included.

## Conflicts of Interest

The authors declare no conflicts of interest.

## Data Availability

Data sharing is not applicable to this article as no new data were created or analyzed in this study.
